# Foods, Fats, and Feeding

**Published:** 1916-06-03

**Authors:** 


### FOODS, FATS, AND FEEDING.

Advisers on the food question are with us always and the war has given them a great opportunity. What we should or should not eat and drink has been proclaimed to us by peers of the realm both reverend and lay, as well as by many mortals of lesser renown. In some instances the advice claims a moral or patriotic sanction; in others it professes a physiological basis. But the ordinary man, at least so long as he is in good health, settles his food programme on custom and practice and not on chemical reactions or laboratory tests. With, as in present circumstances, the need for economy before his eyes, he recognises that excesses in the direction of luxury may be diminished or greatly reduced. In other words, a certain rough common-sense rather than a scientific calculation is taken as a guide to food expenses, whether this has to satisfy the luxuries of peace time or to content the narrower budget demanded by the war. This latter state of affairs doubtless produces some inconvenience, but to date, at all events, it cannot in this country have produced any appreciable amount of suffering. Restriction is practised voluntarily and under the sense that the wants of the country make it unseemly that money should be spent on sensuous pleasure when so many of our fellow-countrymen are standing at the peril of their lives in defence of our liberties and homes. The way to effect this end is perfectly familiar to most of us and we can dispense with the faddists of the newspapers.

### The Food Question in Germany.

Another aspect of the food question, and one which we can contemplate with more philosophy, is the shortage of supplies alleged to affect our principal enemy. For the moment the announcement is made with great confidence, and we have no more idea than our readers how long it will be necessary to wait for contradiction on the one hand or for confirmation on the other. Still, it may be a pleasing enterprise to contemplate the misfortunes of those who would fain harm us, and in any event such a proceeding may remind us that national blunders and miscalculations are not peculiar to ourselves. At the moment we have the advantage of a study by Sir James Crichton Browne of the food position as it is believed to exist in Germany. What Sir James has to say on any question we always hear with pleasure. We know that throughout his long experience he has gathered a considerable store of common-sense, and that, while he is always in contact with scientific teaching, he checks and controls this by practical knowledge of men and affairs. If he has a weakness—we barely suggest the possibility—it is perhaps a desire to make our flesh creep at the sight of what may happen should we fail to follow his advice. On the present occasion this foible is rather an advantage than otherwise, for the ills he prophesies are not for ourselves but for our foes, and these will in all probability not hear Sir James's voice and even if they did they would not heed it.

### Fat Used for High Explosives.

Sir James's patriotically cheerful conclusions proceed from the view that the Germans are using all the fat they can get for the manufacture of high explosives, and that consequently some millions of people are undergoing a slow fat-starvation. As was to be expected he pays no heed to the amateur efforts which have announced in exaggerated and melodramatic terms the consequences of this state of affairs. But as a physician and a physiologist he knows that fats may not be excluded from the scheme of a nation's diet with impunity, and he tells us of results which, if not certain, are at least within the range of possibility. Sir James recognises the special disaster which absence of fat must bring to a race with "a strong tendency to obesity." These heavy, stout, stolid, fat-consuming men will feel in a special measure the absence of their favourite food, and Sir James's anticipation is that the consequences will be much more widespread than a mere prejudice to personal nutrition. In this, at all events in principle, he is on well-established ground, for he rejects the narrow view that the selection of a nation's food can be based upon effects which can be appreciated by weighing and measuring the individual and by estimating the work produced in foot-pounds and relative to the cost of the intake. Whether in the particular direction on which he condescends Sir James will prove correct time alone can show.

### After-War Effects.

What he looks forward to, if fat continues to be given to explosives rather than to men, is the development in the Berlin rabble of "a lean and hungry look" associated with behaviour which will cause the Kaiser to exclaim, like Julius Cæsar, "Let me have men about me that are fat." So much for the immediate future, and the anticipation commands the goodwill of all of us. But there are further consequences, good for ourselves and bad for the other side. "When the war is over"—we would fain have had a more precise oracle—the long-continued food deficiency must have an enduring harmful effect on the working power of the German people, whereas our own kith and kin, never so well fed as they are now, will be splendidly equipped for the industrial contest. We do not bind ourselves to endorse all Sir James's pictures of what absence of fat from the food of our enemies is going to cause—"crimes, vices, lawlessness, ill-temper, despair, lust, and cruelty." But we are sure that he has dealt with the situation in a broad and philosophic spirit, and has recognised that in food and feeding we have to deal with more subtle influences than balances and chemical tests can determine. The alteration of the diet of a people, whether voluntarily or under compulsion, may have, as he very wisely says, "an influence we do not even now appreciate."

